# The Heat Shock Protein 27 Immune Complex Enhances Exosomal Cholesterol Efflux

**DOI:** 10.3390/biomedicines8080290

**Published:** 2020-08-17

**Authors:** Chunhua Shi, Daiana Alvarez-Olmedo, Yuan Zhang, Badal S. B. Pattar, Edward R. O’Brien

**Affiliations:** Health Research and Innovation Center, Libin Cardiovascular Institute, University of Calgary, Cumming School of Medicine, Calgary, AB T2N4Z6, Canada; cshi3@mdanderson.org (C.S.); daiana.alvarezolmedo@ucalgary.ca (D.A.-O.); yuan9@ualberta.ca (Y.Z.); badal.pattar@ucalgary.ca (B.S.B.P.)

**Keywords:** exosome, Heat Shock Protein 27, antibody, immune complex

## Abstract

Previously, we demonstrated that Heat Shock Protein 27 (HSP27) reduces the inflammatory stages of experimental atherogenesis, is released by macrophage (MΦ) exosomes and lowers cholesterol levels in atherosclerotic plaques. Recently, we discovered that natural autoantibodies directed against HSP27 enhance its signaling effects, as HSP27 immune complexes (IC) interact at the cell membrane to modulate signaling. We now seek to evaluate the potential role of the HSP27 IC on MΦ exosomal release and cholesterol export. First, in human blood samples, we show that healthy control subjects have 86% more exosomes compared to patients with coronary artery disease (*p* < 0.0001). Treating human THP-1 MΦ with rHSP27 plus a validated anti-HPS27 IgG antibody increased the abundance of exosomes in the culture media (+98%; *p* < 0.0001) as well as expression of Flotillin-2, a marker reflective of exosomal release. Exosome cholesterol efflux was independent of Apo-A1. THP-1 MΦ loaded with NBD-labeled cholesterol and treated with the HSP27 IC showed a 22% increase in extracellular vesicles labeled with NBD and a 95% increase in mean fluorescent intensity. In conclusion, exosomal abundance and secretion of cholesterol content increases in response to HSP27 IC treatment, which may represent an important therapeutic option for diseases characterized by cholesterol accumulation.

## 1. Introduction

Extracellular vesicles (EVs), characterized as a heterogenous group of microparticles, play an important role in intercellular communication [1]. EVs differ in size, density, protein and lipid composition, as well as the pathways by which they are released [1,2,3]. The focus of our previous studies was a discrete population of EVs known as exosomes-particles that are considered to be between 30–150 nm in diameter [1,4,5] and with a density of 1.13–1.19 g/mL on a sucrose gradient [6,7]. Exosomes have both a unique protein and lipid composition (e.g., tetraspanins such as CD81, the abundance of raft-lipids such as cholesterol and heat shock proteins (HSPs) [8]), as well as a diverse load of cargo, varying from proteins, nucleic acids and other cellular materials that play a role in paracrine signaling [9].

Several studies implicate exosomes in a spectrum of physiological and pathological processes [10]. Nevertheless, the factors that determine the biogenesis, composition, release and potentially the function of exosomes are incompletely understood. Exosome biogenesis occurs by inward budding of late endosomes or multivesicular bodies via processes known as microautophagy [11], macroautophagy and/or chaperone-mediated autophagy [9]. The lateral segregation of endosomal cargo can be mediated by pathways dependent and independent of the endosomal sorting complex required for transport [12,13,14]. Lipid rafts, which are cell membrane domains enriched in cholesterol, sphingolipids, glycosylphospatidylinositol-anchored proteins and other lipid raft-associated proteins, may also be involved in exosome generation. Moreover, incorporation of raft-associated proteins and molecules such as, gangliosides, major histocompatibility complex class II molecules, Flotillin-1 or Flotillin-2 (which acts as membrane platforms for regulating signal transduction, and may be required for exosome release), may facilitate exosomes assembly [15]. In addition to purposeful signaling roles, exosome secretion serves as a means of disposal for unwanted proteins and lipids [16]. This is particularly relevant to cardiovascular diseases, as exosomes contain around four times higher amounts of cholesterol compared to cells, and therefore may be involved in cholesterol homeostasis.

Heat shock protein 27 (HSP27) is a member of the small HSP protein family and is involved in a variety of biological activities including ATP-independent molecular chaperoning, prevention of apoptosis, and more recently attenuating atherogenesis. Our laboratory reported that reduced serum HSP27 levels are associated with cardiovascular disease [17,18], and along with four independent laboratories demonstrated reduced arterial HSP27 expression with atherosclerosis [19,20,21,22]. Conversely, we showed that increasing HSP27 serum levels, by systemic over-expression, bone marrow transplantation or administration of the recombinant protein, not only reduced plaque burden, but also correlated with a lower tendency for experimental plaque rupture [23,24]. While an important feature of these experiments was the observation of reduced cholesterol accumulation in the artery wall of HSP27 treated mice, the precise nature by which HSP27 reduces plaque cholesterol content is incompletely understood. 

Previously, we showed that HSP27 is found in extracellular vesicles, and using transmission electron microscopy demonstrated that it specifically localizes to the exosomal membrane [25]. Moreover, HSP27-laden exosomes activate the NF-κB pathway to promote the release of IL-10 from THP-1 MΦ. In vitro, we showed that recombinant HSP27 (rHSP27) upregulated the expression of the transporters ABC-A1 and ABC-G1 that are involved in reverse cholesterol transport (RCT). Although rHSP27 therapy in vivo resulted in modest reductions in plaque cholesterol content, these studies were characterized by only a ~10% increase in facilitated cholesterol efflux. Hence, we now seek to explore if HSP27 may mediate RCT by other means, looking specifically at exosomal cholesterol transport. To study the in vitro role of exosomal HSP27 we will incorporate new information showing that anti-HSP27 IgG antibodies levels are more abundant in healthy subjects vs. CVD patients. To this end, we have generated and validated a polyclonal anti-HSP27 IgG antibody (PAb) that combines with HSP27 to form an immune complex (IC) that facilitates the biological effects of HSP27 (referred to as Immune Complex Altered Signaling and Transport (ICAST)) [18,26].

## 2. Experimental Section

### 2.1. Patient Information

Human blood samples were obtained from healthy young volunteers (4 males, mean age 31.8 years) and patients with coronary artery disease (CAD; 2 males, 2 females, mean age 58.5 years) according to a protocol approved by the Ottawa Heart Institute Research Ethics Committee where this work was originally performed. The investigational plan conformed with the principles outlined in the Declaration of Helsinki for use of human tissue or subjects.

### 2.2. Cell Culture and Treatment

The THP-1 human monocytic cell line (TIB-202; ATCC, Manassas, VA, USA) was maintained and cultured in RPMI-1640 medium 11875093 (Thermo Fisher-Gibco, Waltham, MA, USA), and supplemented with 10% FBS, penicillin/streptomycin, and sodium pyruvate (1 mM) as described previously [27]. Cells were plated at a density of 1 × 10^6^ cell/mL, and treated with 50 ng/mL of phorbol myristate acetate (PMA, Sigma, St. Louis, MO) for 48 h to differentiate into MΦ, as described previously [28]. Thereafter, the cells were cultured in fresh media without PMA for 24 h, then treated with: 1 µg/mL of recombinant HSP27 (rHSP27), C1 (1 µg/mL, rC1) alone, as well as either rHSP27 or rC1 (1 µg/mL) plus the anti-HSP27 IgG antibody (PAb, 5 µg/mL).

### 2.3. Recombinant Protein Preparation

The recombinant proteins were prepared as previously described [27]. Briefly, N-terminal His-tagged full-length rHSP27 and N-terminal truncated mutant rC1 (AA90–205) compliment (c)-DNA were constructed in a pET-21a vector, and the plasmids transformed in an *Escherichia coli* expression strain Rosetta™DE3 (Novagen, MilliporeSigma; Billerica, MA, USA). Recombinant proteins were purified with a Ni-NTA resin and Q-Sepharose™ (GE Healthcare; Chicago, IL, USA), followed by refolding via dialysis. Endotoxin was removed with High-Capacity Endotoxin Removal Resin (Pierce, Thermo Fisher Scientific; Waltham, MA, USA), and the purity of the proteins were determined by SDS-PAGE (purity > 99%). The endotoxin concentration was lower than 2 EU/mg protein, as assessed by the Limulus Amebocyte Lysate PYROGENT™ 125 Plus assay (Lonza; Basel, Switzerland).

### 2.4. Preparation of HSP27 Polyclonal Antibody

A rabbit polyclonal IgG antibody mimicking human HSP27 autoantibody was produced according to the standard procedure outlined by Cedarlane Laboratories (Ontario, Canada) that align with the requirements of the Canadian Council on Animal Care. The detailed summary of the methods used to generate and validate this PAb are available elsewhere [18].

### 2.5. Exosome Purification

Exosomes derived from THP-1 MΦ or human serum samples were collected and subjected to sequential centrifugation as previously described [25]. Briefly, sequential low-speed centrifugation (2000*g* × 10 min and 10,000*g* × 30 min) were performed, and in each case, the pellet (with floating cells and cellular debris) was discarded and the supernatant retained. The supernatant was further refined using a 100 kDa MW cutoff centrifuge filter (Millipore Sigma), then ultra-centrifuged at 100,000*g* × 90 min. The pellet was resuspended in medium or Dulbecco’s phosphate-buffered saline (PBS) to yield a purified exosome fraction.

### 2.6. Flow Cytometry Analysis

The THP-1 was loaded with fluorescent NBD cholesterol [22-(N(-7-nitrobenz-2-oxa-1,3-diazol-4-yl)amino)-23,24- bisnor-5-cholen-3β-ol] (Thermo Fisher Scientific), then washed with PBS and treated with rHSP27, rC1 or the HSP27 IC for 16 h, as described previously [29]. The purified exosome fraction was incubated with anti-human CD81 mAb (349502; BioLegend; San Diego, CA, USA) followed by anti-mouse IgG PE-Texas Red. Fluorescence-activated cell sorting (FACS) analysis was performed with an LSRII Flow Cytometer (BD Biosciences, San Jose, CA, USA) at FITC-488 nm (for NBD cholesterol) and PE-532 nm lasers (for DC-81 PE-Texas red). Each experiment quantified: (a) the percentage of extracellular vesicles that were fluorescent, and (b) the mean fluorescence intensity (MFI) which represents the amount of NBD cholesterol present for particular experimental conditions.

### 2.7. Exosomal HSP27 ELISA Detection

Exosomes derived from THP-1 or human serum samples were detected using an ELISA, described previously [25]. Briefly, anti-CD81 antibodies (BioLegend) were coated onto 96-well plates and used to capture exosomes from 100 mL of purified exosomal fractions, supernatant fractions or cell culture media. Anti-MHC-II was conjugated with biotin (EZ-Link Sulfo-NHS-LC-LC-Biotin, 21338; Thermo Fisher Scientific; Waltham, MA, USA) and was used as a detection antibody with peroxidase-conjugated streptavidin (016-030-084; Jackson ImmunoResearch Laboratories) plus the SuperSignal ELISA Femto Maximum Sensitivity Substrate (37074; thermos Fisher Scientific), according to the manufacturers’ instructions. Signal quantitation was performed using a microplate reader at 450 nm (BioTek’s Synergy™ Mx, Winooski, VT, USA). The linearity of the standard curve for detecting exosomes was evaluated (data not shown), as well as the loading amount and the precision of the method (coefficient of variation (CV) intra-assay ≤10%). The reagent blank used for the first study was complete medium, which gave a higher background, potentially due to the contribution of plasma-derived exosomes (limit of detection 0.202). Therefore, for all subsequent ELISA experiments, the pellet was resuspended in PBS and PBS was used as a blank (limit of detection 0.134).

### 2.8. Fast Protein Liquid Chromatography (FPLC)

To trace the passage of molecules according to their dimensions, size exclusion chromatography using an AKTA Primer Plus fast protein liquid chromatography system (GE Healthcare) with Superose™ 6 10/30 GL Column (GE Healthcare) was employed. Samples were diluted in PBS to 20 μg/mL, and the PAb was mixed with rHSP27 or rC1 for 30 min. Aliquots of 0.2 mL per sample were loaded into each column and the fractions were eluted with 0.2 mL/min PBS buffer with the absorption spectra monitored at 280 nm. The mix of Blue Dextran (2000 kD), Apoferritin (443 kD), Alcohol Dehydrogenase from yeast (150 kD) and bovine serum albumin (66 kD; Sigma) were employed as the protein standards. The reaction lasted 24 h, and 10 µg/mL of Apo-A1 was used in the Apo-A1-Media containing THP-1 MΦ. 

### 2.9. Immunoblotting Analysis

After treating with the various reagents, THP-1 MΦ were washed with PBS, and lifted by pipette with 1 mL of PBS and treated with lysis buffer (50 mM HEPES, 0.5 M sodium chloride, 1.5 mM magnesium chloride, 1 mM EDTA, 10% (*v*/*v*) glycerol, 1% Triton X-100, and Protease Inhibitor Cocktail (Roche, MilliporeSigma; Billerica, MA, USA)). Protein concentration was determined by Bradford’s micromethod (Sigma-Aldrich; Darmstadt, Germany), according to the manufacturer’s protocol. Whole protein (50 µg) from each treatment group was loaded onto a 10% SDS-PAGE gel and transferred to a polyvinylidene fluoride membrane using the iBlot^®^ dry blotting system (Life Technologies, New York, NY, USA) 7 min program according to the manufacturer’s protocol. Membranes were then subjected to western blotting using the following antibodies: polyclonal rabbit anti-Flotillin-2 (Cedarlane, 1:1000; Burlington, ON, Canada) or mouse anti-beta actin antibody (NOVUS Biologicals, 1:10,000; Littleton, CO, USA) followed by incubation with the secondary antibody conjugated with peroxidase (Abcam; 1:10,000; Cambridge, UK) before being incubated with the enhanced chemiluminescent Amersham detection reagent, according to manufacturer’s recommendations. Visualization was performed using the ChemiDoc Imaging System (Bio-Rad; CA, USA) and densitometric analysis was measured with ImageJ analysis software (National Institutes of Health (NIH); Bethesda, MD, USA).

### 2.10. Statistical Analysis

Bar graph data were generated with GraphPad Prism version 7.00, (GraphPad Software, La Jolla, CA, USA), and expressed as means ± SEM. Statistical comparisons were carried out using one-way ANOVA analysis of variance followed by Tukey’s multiple comparison tests, unless otherwise indicated. The results were considered statistically significant at *p* < 0.05.

## 3. Results

### 3.1. Serum Exosome Levels were Lower in CAD Patients Compared to Healthy Control Subjects

**Figure 1:**
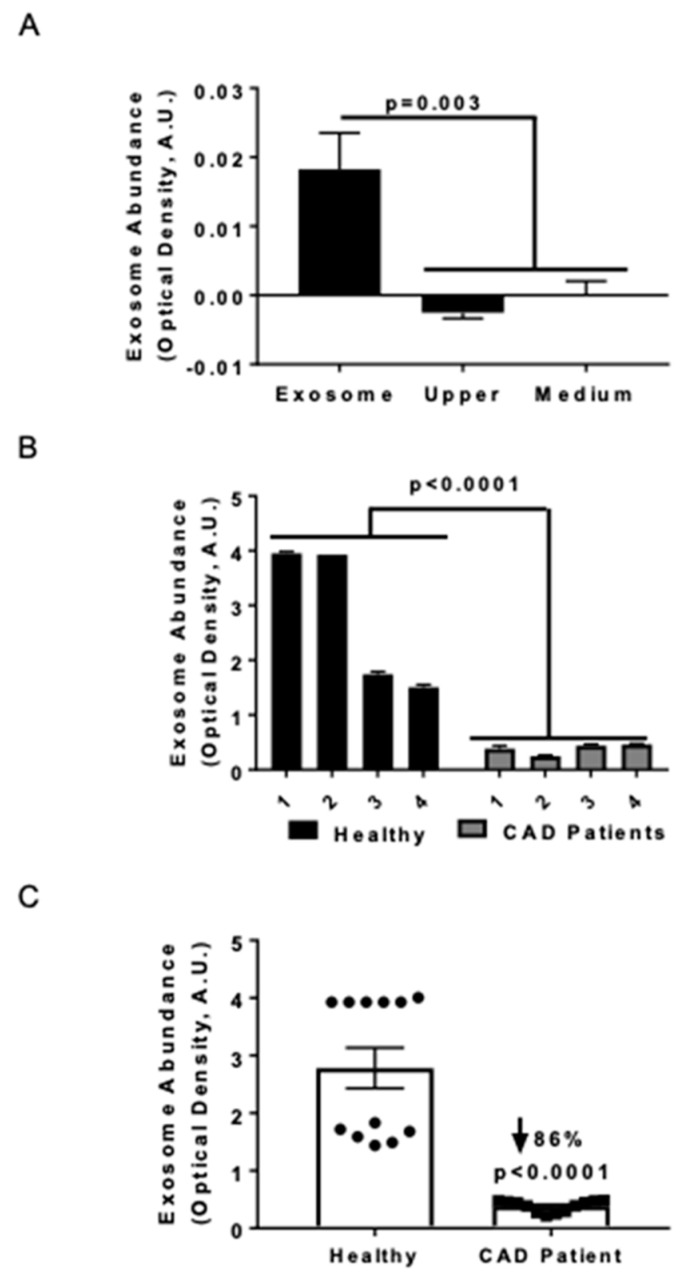
Serum exosome levels in coronary artery disease (CAD) patients are diminished compared with healthy controls. Exosome abundance was assessed by ELISA and quantified by optical density using arbitrary units obtained after background subtraction (A.U.). (**A**) ELISA performed with anti-MHC primary (capture) antibody and anti-CD81 antibody to detect exosomes in the purified fractions (*n* = 4) from THP1 MΦ. Horizontal axis labels: Exosome = Purified exosome fraction; Upper = the upper fraction without exosomes after ultracentrifugation; Medium = cell culture medium (only) as negative control. (**B**) Detection of serum exosomes in CAD patients and healthy (control) subjects. Anti-CD81 was applied as the capture antibody and anti-MHC-II was used as the developing antibody to quantify exosomes in each sera sample. Analysis was performed via two-way ANOVA (*n* = 4 subjects per group, with each subject’s sample measured in triplicate). (**C**) Detection of exosomes in the sera of CAD patients and healthy individuals (control). Anti-CD81 was applied as the capture antibody and anti-MHC-II was used as the developing antibody to quantify exosomes in each sera sample. The data were analyzed using a Mann-Whitney test and plotted to note the exosome distribution in each population. Exosome levels were downregulated in CAD patients.

Previously we noted that serum HSP27 levels were higher in health vs. cardiovascular disease [18,23], and now sought to assess exosome abundance in two small human cohorts. Exosomal abundance was quantified using an ELISA method that consisted of an anti-CD81 antibody to capture exosomes, followed by immunolabeling with an anti-MHC-II antibody [25]. In order to validate the purification method, the exosome abundance was measured in the supernatant (upper fraction) and the purified exosome fraction (obtained by resuspending the pellet in medium (Figure 1A) or PBS for all subsequent studies). No quantifiable exosome signal was found in the upper fraction following ultracentrifugation, and the medium was designated as the negative control (Figure 1A). The exosomal marker MHC-II was exclusively detected in the purified exosomal fraction and not in the supernatant fractions. The precision of the measurements was evaluated in both the exosomal abundance–concentration curve (data not shown) and serum samples; the values obtained from four CAD patients and four healthy donors were compared (Figure 1B). Combining the exosomal levels of each individual and comparing the two groups, we noted 86% fewer exosomes in CAD patients compared to healthy controls (Figure 1C; *p* < 0.0001).

### 3.2. HSP27 IC Increases Exosomal Release and Flotillin-2

**Figure 2:**
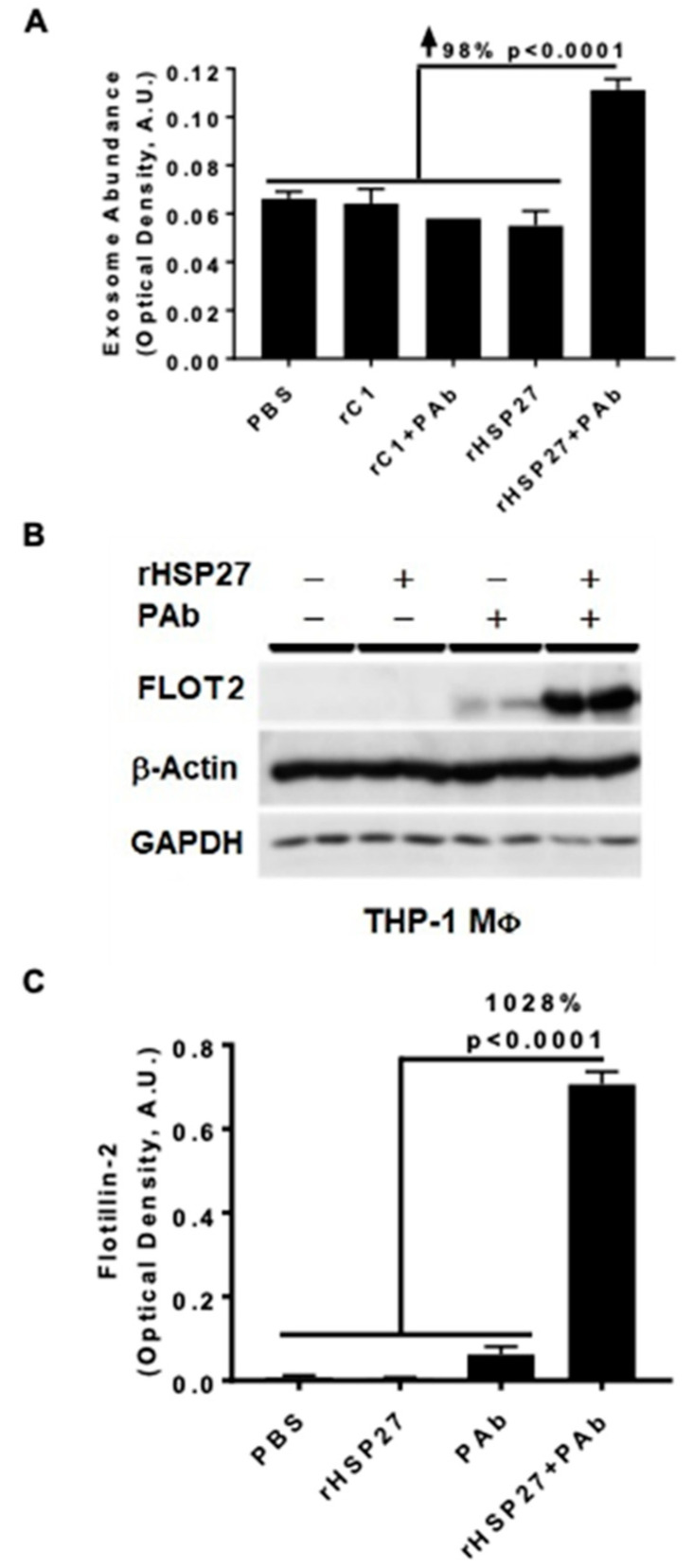
HSP27 IC Modulate Cholesterol Efflux and Expression of FLOT2. (**A**) MΦ exosome release was assayed in the media by ELISA. An anti-CD81 antibody was used to capture exosomes and anti-MHC-II/biotin antibody was used to detect the secondary exosomal biomarker (MHC-II). The HSP27 IC increased the release of exosomes by 98% compared to rC1 + PAb (*p* < 0.0001). (**B**) Western blot analysis of Flotillin-2 (FLOT2) expression in THP-1 MΦ after 24 h treatment with PBS, rHSP27, PAb or rHSP27 + PAb, as indicated. (**C**) Densitometry quantification of the FLOT2/β-actin. FLOT2 expression was enhanced by 1028% after treatment with the HSP27 IC (only). Each band was measured with ImageJ, and the data set was analyzed via one-way ANOVA. The bars represent the means of three independent experiments ± SEM (*p* < 0.0001).

In atherosclerotic plaques, HSP27 expression is primarily found in MΦ [23]. To explore if HSP27 alone or combined with the anti-HSP27 PAb alters exosomal release in THP-1 MΦ, we used the ELISA method with an anti-CD81 antibody to capture exosomes and a biotinylated anti-MHC-II antibody to detect the secondary exosomal biomarker (MHC-II). PBS was used as a mock control treatment, representing the basal levels of exosomes obtained at the standard growth conditions without stimulation. All treatments, including rHSP27 alone had no effect on exosomal abundance; however, the HSP27 IC increased the number of exosomes by 98% compared to the control [rC1 + PAb] (Figure 2A; *p* < 0.0001).

Next, we assessed the impact of the HSP27 IC on the expression of the exosomal marker flotillin-2 (FLOT2) in THP-1 MΦ treated with rHSP27 alone or with the PAb. Using the same exosomal capture ELISA we noted that relative to controls [rHSP27 + PAb] increased FLOT2 expression by 1028% (*p* < 0.001; Figure 2B,C).

### 3.3. Exosomal Cholesterol Export is Independent of Apo AI or HDL

**Figure 3:**
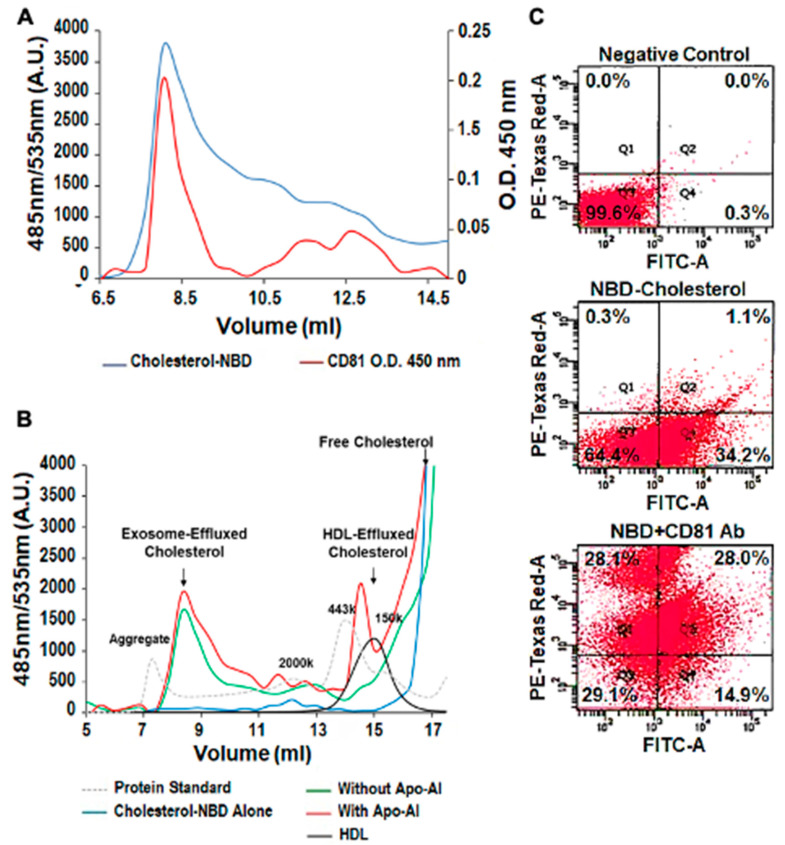
Cholesterol can be Exported in Exosomes Independent of Apo-A1. (**A**) Analysis of NBD cholesterol in exosome fraction by fast protein liquid chromatography (FPLC) and ELISA. The exosome fraction was loaded into the FPLC and the signal of NBD cholesterol was recorded by its fluorescent signaling (485 nm/535 nm). CD81 was measured with the ELISA method. This involved each fraction being coated on an ELISA plate and the anti-CD81 monoclonal antibody was used to detect its concentration (O.D. 450 nm). (**B**) NBD cholesterol was transported outside the cell in both the HDL and exosome fractions, but did not require Apo-A1, thereby indicating that both pathways are used for cholesterol efflux. HDL alone and NBD cholesterol in the media were used as the controls. Protein standards were loaded to indicate each fraction’s molecular weight. 2000 kDa = 20 nm diameter. (**C**) Colocalization of NBD cholesterol (FITC signal) and CD81 (PE-Texas Red-A), using flow cytometry in purified exosome samples derived from THP-1 MΦ. The exosome fractions were then scanned by flow cytometry detecting NBD cholesterol (FITC). The experiments were performed in triplicate. Representative graph of the EVs percentage immunolabeled for: (i) background control (0.0%), (ii)) NBD cholesterol (1.1%) and (iii) CD81 (28.0%). NBD cholesterol and the exosome biomarker CD81 colocalized by both FPLC separation and flow cytometry techniques. The results confirm that cholesterol can be transported out of the cell, through exosomal pathways, in the presence and absence of HDL and Apo-A1.

NBD cholesterol and CD81, the exosome biomarker, were used to follow THP-1 MΦ cholesterol export in exosomes via FPLC and flow cytometry. Both FPLC separation and flow cytometry techniques confirmed that cholesterol can be exported out of the cells in a pattern that follows that of exosomes. For example, the FPLC data show partial superimposition of the size characterization curves for NBD cholesterol and CD81 (Figure 3A). The exosomal cholesterol efflux pattern was independent of Apo-A1 (Figure 3B, left arrow) but in the presence of Apo-A1 mimicked that of HDL (Figure 3B, right arrow). Finally, using flow cytometry we observe colocalization of NBD cholesterol (FITC signal) and the exosomal marker CD81 (PE-Texas Red-A) with up to 28% of microparticles being positive for both markers (Figure 3C).

### 3.4. HSP27 Immune Complex Increases Exosomal Cholesterol Efflux in THP-1 MΦ

**Figure 4:**
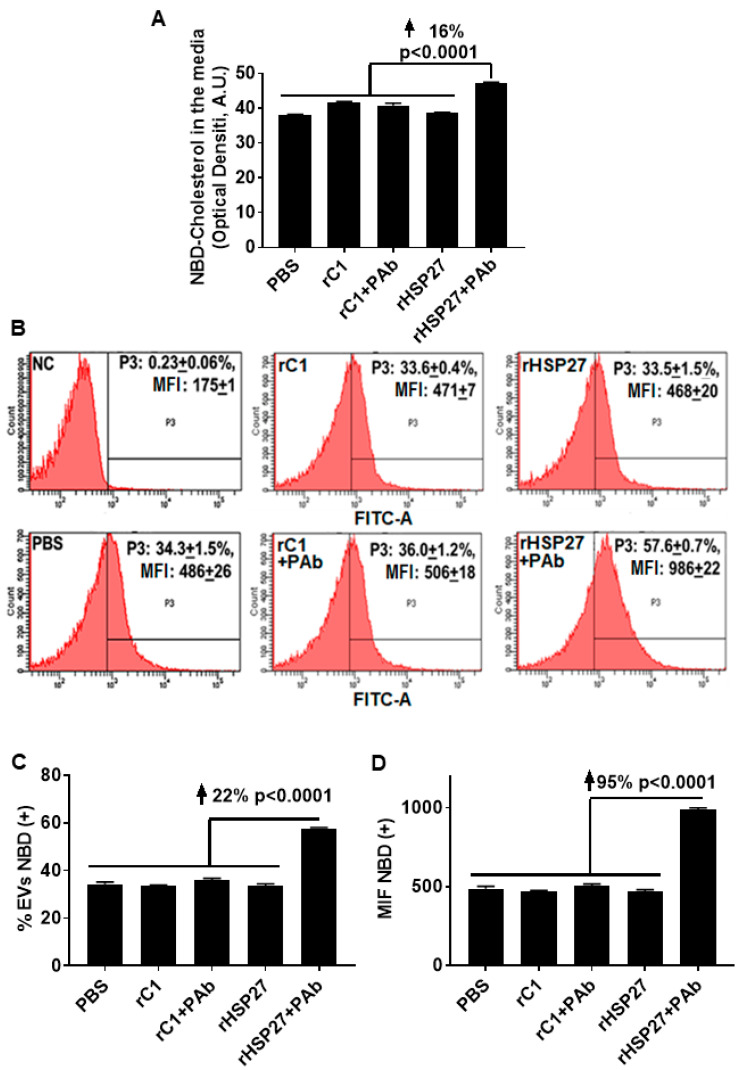
HSP27 IC Modulates Cholesterol Efflux Via Exosomes. (**A**) Effects of rHSP27 alone or the HSP27 IC on cholesterol efflux in THP-1 MΦ. NBD cholesterol assayed in the media. The HSP27 IC increased NBD cholesterol efflux to the medium by 16% after 24 h of incubation (*p* < 0.0001). (**B**) Fluorescence-activated cell sorting (FACS) analysis of NBD cholesterol secretion through extracellular vesicles from THP-1 MΦ. The medium was centrifuged for 30 min at 10,000*g* × and the supernatant was used for FACS. The exosome fractions were then scanned by flow cytometry to the NBD cholesterol signal (FITC). The graphs are representative of 3 separate experiments: (i) NC = negative control (0.23 +/− 0.06%), (ii) rHSP27 (33.5 +/− 1.5%), (iii) rC1 (33.6 +/− 0.4%), (iv) PBS (34.3 +/− 1.5%), (v) rHSP27 + PAb (57.6 +/− 0.7%) and rC1 + PAb (36.0 +/− 1.2%). (**C**) Statistical analysis of the percentage of extracellular vesicles positive for NBD cholesterol. The HSP27 IC increased the number of NBD cholesterol positive extracellular vesicles by 22% (*p* < 0.0001), in comparison to the control treatment [rC1 + PAb]. (**D**) Statistical analysis of mean fluorescente intensity (MFI; measured as NBD cholesterol). Treatment with the HSP27 IC resulted in a 95% increase in the amount of NBD cholesterol transported by the extracellular vesicles (*p* < 0.0001 vs. [rC1 + PAb]).

To assess the potential for the HSP27 IC to alter cholesterol export, NBD loaded THP-1 MΦ were treated with the indicated drugs, then NBD cholesterol was evaluated in the media and in the exosome fractions. Only [HSP27 + PAb] treatment increased the amount of NBD cholesterol in the media by 16% (*p* < 0.0001; Figure 4A), without changes in NBD cholesterol content with other treatments, including HSP27 (alone). Using flow cytometry, the analysis showed an increase in the abundance of NBD cholesterol positive extracellular vesicles by 22% comparing the HSP27 IC to [rC1 + PAb] (Figure 4B,C). Moreover, this augmentation in NBD cholesterol resulted in an overall increase in MFI of 95% compared to [rC1 + PAb] (Figure 4D), thereby suggesting that the exosomes were heterogeneous in transporting NBD cholesterol, with 78% of the extracellular vesicles not participating, but those that did being very heavily laden with NBD cholesterol.

## 4. Discussion

**Figure 5:**
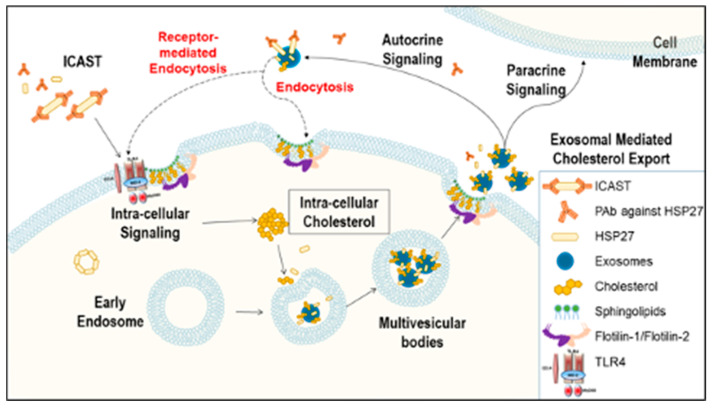
Working model for HSP27 Immune Complex Altered Signaling and Transport (ICAST) modulation of cholesterol efflux. The HSP27 Immune Complex Altered Signaling and Transport unit (or exosomes containing HSP27 + anti-HSP27 antibody) trigger intracellular signaling that modulates the release of cholesterol loaded exosomes. For example, expression of Flotillin-2 is increased, and the cholesterol loaded into exosomes, as well as the number of exosomes released is enhanced. Once the exosomes are released to the extracellular space, they may act via autocrine or paracrine signaling, or proceed to cholesterol disposal via yet to be elucidated mechanisms.

Recently, our laboratory demonstrated that healthy individuals, compared to CAD patients, have not only higher HSP27 blood levels, but also increased levels of natural IgG autoantibodies to HSP27 [18]. Moreover, we noted that in the extracellular space the HSP27 IC can alter cell signaling, acting at the cell membrane and also undergoing internalization [18]. Although we previously showed that HSP27 is released from MΦ in exosomes, we are only now beginning to understand the significance of exosomal HSP27–including its role as part of the HSP27 IC. While one might postulate that these anti-HSP27 antibodies would undermine the biological effects of HSP27, by masking the protein and preventing it from performing its extracellular functions, just the opposite appears to be true. In fact, our recent observations form the basis of a revised working model: HSP27 Immune Complex Altered Signaling and Transport (ICAST; Figure 5).

In healthy individuals, exosomes are regularly released by a variety of cells, including endothelial and smooth muscle cells, platelets, cardiomyocytes, and stem cells. Thus, exosomes are purported to be an integral part of the intercellular communication system that may either maintain health or, when dysregulated, contribute to a variety of disease processes. Indeed, extracellular vesicles play crucial roles in the development and progression of atherosclerosis [30]. Hence, it is interesting that, in a small cohort of human subjects, we found exosomal serum levels reduced in patients with CAD (Figure 1).

Lipid intracellular levels can be regulated either by classical pathways (that mediate the efflux of cholesterol and phospholipids to lipid-poor apolipoproteins), or by vesicles. Classical pathways depend upon carrier proteins, such as ABC transporters, phospholipids transfer proteins, fatty-acid-binding proteins and lipoproteins. In addition to proteins and nucleic acids, exosomes can transport lipids, such as eicosanoids, prostaglandins, leukotrienes, fatty acids, cholesterol and lipid-related enzymes. Here we showed that in the presence of Apo-A1, both HDL and exosome fractions transport cholesterol, but in the absence of Apo-A1, only the exosome fraction was able to transport cholesterol (Figure 3B). Therefore, exosomal export may be an important mechanism of removing cholesterol from tissues when HDL levels are reduced [31] or when classical regulation of cholesterol is impaired (e.g., Niemann-Pick type C1 disease, a lysosomal storage disorder leading to abnormal accumulation of free cholesterol and sphingolipids within the late endosomal and lysosomal compartments) [32].

The cellular release of exosomes stems from the displacement of the microvesicle bodies along microtubules, a process partially controlled by the cholesterol content of the cells [33]. In monocytes, exosome internalization triggers cholesterol accumulation into lipid droplets [7], and once MΦ become foam cells, it is believed that they can no longer leave the atherosclerotic plaque. However, it is well known that HSP27 is also able to regulate microtubules organization [34], plus we now show new data on its effects on FLOT2 (Figure 2B,C). Flotillin participates in scaffolding functions and endocytosis, and its two isoforms, Flotillin-1 and Flotillin-2, are enriched in exosomes and can be used to quantify exosomal release [13]. Indeed, Flotillin can recruit cholesterol to the cell and intracellular membranes, where it colocalizes with cholesterol [13]. Hence, our study suggests a new way to modulate lipid export, whereby the HSP27 IC enhances the number of secreted exosomes and the cholesterol levels in the MΦ exosome (Figure 4).

Finally, our study is not without limitations. In particular, the THP-1 MΦ were grown in FBS. Hence, we have not excluded the potential contribution of plasma-derived exosomes to our experiments. Whether plasma-derived exosomes also increased the level of exosome detection is also not clear. However, given that all cells were treated the same, there are significant relative differences between the experimental groups that represent effects that are beyond the potential contribution of plasma-derived exosomes.

## 5. Conclusions

Here we showed in THP-1 MΦ that HSP27 IC enhances both the number of secreted exosomes and the cholesterol concentrations in exosomes, thereby illustrating the potential importance of HSP27 immunotherapy for atheroprotection—perhaps both as a preventative measure as well as reducing plaque cholesterol content after it has formed. Although more research is still required to be applicable to the clinic, HSP27-laden exosomes are promising drug delivery candidates that can communicate between an intracellular compartment of a donor cell and its target cells for diseases that involve cholesterol accumulation.
